# Diet quality assessed by healthy eating index and its association with metabolic aging: mediation by systemic immune-inflammation index

**DOI:** 10.3389/fnut.2026.1836489

**Published:** 2026-05-28

**Authors:** Xinjing Cui, Hao Sun, Zongyao Li, Hua Yu, Mengmeng Wang, Yuantao Qi

**Affiliations:** 1 Qingdao Hospital, University of Health and Rehabilitation Sciences (Qingdao Municipal Hospital), Qingdao, China; 2 Xiyuan Hospital, Chinese Academy of Traditional Chinese Medicine, Beijing, China; 3 Shandong Cancer Hospital and Institute, Shandong First Medical University and Shandong Academy of Medical Sciences, Jinan, China

**Keywords:** AIP, HEI, HOMA-IR, mediation analysis, metabolic aging, SII

## Abstract

**Background:**

Metabolic aging is characterized by insulin resistance, adverse lipid metabolism, and chronic low-grade inflammation, and is associated with higher risks of type 2 diabetes, cardiovascular disease, and mortality. Although diet quality may be related to these processes, evidence on the statistical role of systemic inflammation and the reproducibility of the association in a non-U. S. clinical setting remains limited.

**Objective:**

To examine the association between diet quality, assessed by the healthy eating index-2015 (HEI), and metabolic aging markers, and to evaluate whether the Systemic Immune-Inflammation Index (SII) statistically partially accounts for this association in NHANES and in an external hospital-based cohort from China.

**Methods:**

We conducted a cross-sectional analysis of 15,314 adults aged ≥20 years from NHANES 2005–2020 and an external hospital-based cohort of 833 adults who underwent health examination in Shandong, China, during 2024–2025. Metabolic aging was assessed by Homeostasis Model Assessment of Insulin Resistance (HOMA-IR) and the Atherogenic Index of Plasma (AIP). SII was calculated as platelet × neutrophil/lymphocyte counts. Multivariable linear regression, restricted cubic spline analyses, and statistical mediation analyses were performed. In the external cohort, HEI-2015 was used as a harmonized diet-quality metric to reproduce the same exposure definition, while acknowledging that it does not fully capture culture-specific dietary patterns.

**Results:**

Higher HEI scores were inversely associated with HOMA-IR (*β* = −0.70, 95% CI: −1.10 to −0.30, *p* < 0.01) and AIP (*β* = −0.06, 95% CI: −0.08 to −0.04, *p* < 0.01) in NHANES. SII statistically accounted for 7.3 and 10.5% of the observed associations for HOMA-IR and AIP, respectively. Similar inverse associations were also observed in the external cohort, where the mediated proportions were larger.

**Conclusion:**

Higher diet quality was associated with lower metabolic aging markers, and SII statistically partially accounted for these associations. The external cohort reproduced the direction of the associations in a distinct Chinese clinical setting, but cultural differences and the cross-sectional design warrant cautious interpretation.

## 1 Introduction

Metabolic aging is characterized by a progressive decline in metabolic function, including exacerbated insulin resistance, dysregulated lipid metabolism, and chronic low-grade inflammation, which collectively increase susceptibility to type 2 diabetes, cardiovascular diseases, non-alcoholic fatty liver disease, and mortality (1, 2). With global population aging, the prevalence of metabolic aging-related conditions is rising, posing substantial health, economic, and societal challenges (3–7). The metabolic deterioration associated with aging not only elevates chronic disease risk but also impairs quality of life and contributes to mental health burdens, while increasing healthcare costs and reducing societal productivity (8, 9).

Accumulating evidence highlights dietary patterns as key modulators of metabolic aging (10–12). HEI provides a validated composite measure of diet quality based on adherence to the U. S. Dietary Guidelines, with higher scores reflecting increased intake of fiber, unsaturated fats, vitamins, and minerals, and reduced consumption of refined carbohydrates, trans fats, and added sugars (13, 14). Prior studies have demonstrated inverse associations between HEI and risk of obesity, cardiovascular disease, type 2 diabetes, and metabolic syndrome, suggesting a protective role of high-quality diets (15–18).

Chronic low-grade systemic inflammation is a central mechanism driving metabolic aging, disrupting insulin signaling and lipid homeostasis (19–21). Elevated inflammatory markers, including TNF-*α*, IL-6, and CRP, are strongly associated with impaired insulin sensitivity and dyslipidemia (22, 23). SII, integrating neutrophil, platelet, and lymphocyte counts, offers a robust, composite measure of systemic inflammation and immune status, and has been linked to metabolic dysregulation, cardiovascular disease, and malignancy risk (24–26).

Despite these insights, several gaps remain. First, most available studies have focused on single metabolic outcomes or metabolic syndrome rather than jointly evaluating insulin resistance and lipid-related metabolic aging markers. Second, the extent to which systemic inflammation statistically explains the association between diet quality and metabolic aging remains insufficiently characterized. Third, evidence on whether the direction of these associations can be reproduced in a non-U. S. clinical setting is scarce. Therefore, we analyzed a large nationally representative NHANES sample and then examined the same analytical framework in an independent hospital-based cohort from China. We emphasize that the external cohort was used to test reproducibility of the direction of association in a distinct setting rather than to claim full cross-cultural equivalence of dietary patterns.

## 2 Materials and methods

### 2.1 Study design and sample selection

This study had two components: a primary cross-sectional analysis of NHANES and an external hospital-based validation analysis. The purpose of including the hospital cohort was not to directly compare results across different time periods or populations, but to examine whether the direction and statistical pattern of the association could be reproduced in an independent non-U. S. clinical setting, despite differences in sampling frame and calendar time (NHANES 2005–2020 vs. hospital cohort 2024–2025).

### 2.2 NHANES cohort

For the primary analysis, we used data from the National Health and Nutrition Examination Survey (NHANES) 2005–2020, a nationally representative survey of the non-institutionalized U. S. civilian population approved by the National Center for Health Statistics (NCHS) Research Ethics Review Board. All participants provided written informed consent.

A total of 53,672 participants were initially included. Participants were excluded according to the following criteria: (1) those with incomplete laboratory data (*n* = 29,884); (2) those with incomplete dietary information (*n* = 5,140); and (3) those with incomplete demographic information (*n* = 3,326) or extreme values (*n* = 8). After applying these criteria, a total of 15,314 participants aged ≥20 years were included in the final analysis.

### 2.3 Hospital-based validation cohort

To examine external reproducibility, we assembled an independent hospital-based cohort of adults who underwent routine health examination at the Health Examination Center of the Second Affiliated Hospital of Shandong First Medical University, China, between 2024 and 2025. This cohort was a convenience clinical sample rather than a representative sample of the general Chinese population.

**Figure 1:**
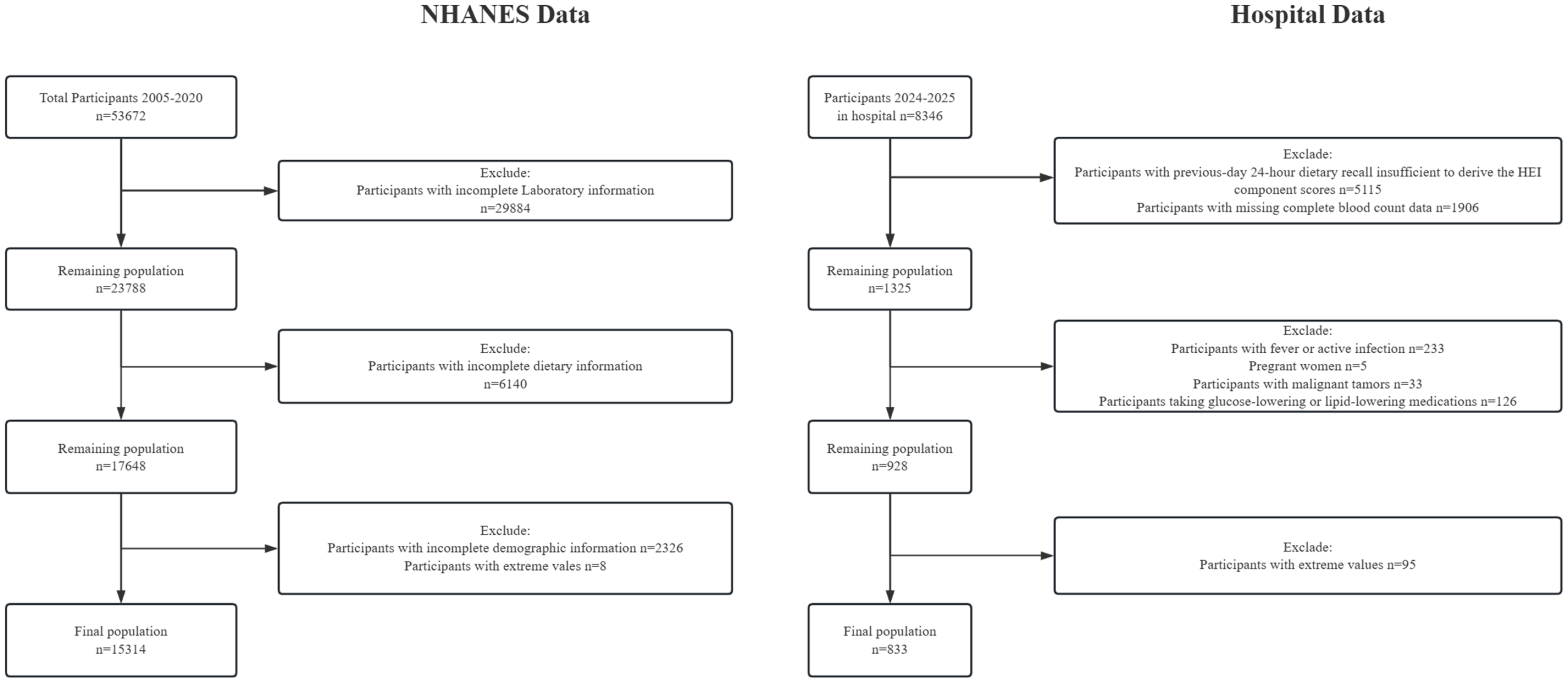
Flowchart of participant selection in U. S. adults from NHANES 2005–2020 and adults undergoing health examination in Shandong, China, 2024–2025.

A total of 8,346 participants were initially enrolled. Available information for the validation cohort included demographic characteristics, lifestyle factors, a previous-day dietary recall sufficient for HEI scoring, and laboratory measurements required to derive SII, HOMA-IR, AIP, non-HDL cholesterol, and serum uric acid. Participants were excluded if the dietary recall was insufficient to derive HEI component scores or if required hematologic or biochemical data were missing. Additional exclusions included fever or active infection (*n* = 233), pregnancy (*n* = 5), malignant tumors (*n* = 33), use of glucose-lowering or lipid-lowering medications (*n* = 126), and extreme values (*n* = 95). After exclusions, 833 participants were included in the final analysis (Figure 1).

### 2.4 Sources of core variables

In this study, HEI-2015 was used as the diet-quality metric. For NHANES, HEI-2015 scores were derived from 24-h dietary recall data in What We Eat in America (WWEIA) using the USDA Food Patterns Equivalents Database (FPED). For the hospital-based validation cohort, dietary intake for the previous day was collected by 24-h recall, and reported foods and beverages were mapped to the same 13 HEI-2015 components to create a harmonized score on an energy-adjusted basis. We used this approach to preserve the same exposure definition across cohorts; however, we acknowledge that HEI-2015 is a U. S.-based index and does not fully capture culture-specific dietary patterns in China.

Laboratory data in NHANES were obtained from standardized serum and whole-blood measurements collected in the Mobile Examination Center. In the validation cohort, complete blood count and biochemical measurements obtained during the health examination were used to derive SII, HOMA-IR, AIP, non-HDL cholesterol, and serum uric acid. The core variable definitions were as follows:

SII was used as an inflammatory marker and calculated as:

SII = Platelet count * Neutrophil count/Lymphocyte count, expressed as 10^9^ cells/μL (27).

Metabolic aging was quantified using validated surrogate biomarkers that reflect key domains of metabolic dysfunction. Specifically, HOMA-IR was used as a marker of insulin resistance (28); AIP, calculated as log (triglycerides/HDL-cholesterol), served as an indicator of lipid-related risk (29). These markers have been widely applied in epidemiological studies as indicators of accelerated metabolic aging.

HOMA-IR was used as an indicator of insulin resistance, which is associated with aging, diabetes, and cardiovascular disease. It was calculated as:

HOMA-IR = Fasting insulin (μU/mL) * Fasting glucose (mmol/L)/22.5 (28).

AIP was used to quantify vascular aging, which is associated with lipid metabolism disorders, cardiovascular disease, and metabolic dysfunction. It was calculated as:

AIP = log_10_ (Triglycerides [mg/dL]/HDL-C [mg/dL]) (29).

### 2.5 Covariates

The following covariates were included in the analyses: age, sex (male/female), marital status (married/partnered, divorced/widowed/separated, never married), obesity (BMI ≥ 30 kg/m^2^), smoking status (never, former, current), alcohol consumption, total energy intake, hypertension status, non-HDL cholesterol, and serum uric acid levels. Alcohol consumption was harmonized into five categories: never (<12 lifetime drinks), former (≥12 lifetime drinks but none in the past year), light (current drinking not meeting the moderate or heavy criteria), moderate, and heavy. In the NHANES-based definition, moderate drinking corresponded to 2 drinks/day for women or 3 drinks/day for men, or binge drinking on 2–4 days/month, whereas heavy drinking corresponded to ≥3 drinks/day for women or ≥4 drinks/day for men, or binge drinking on ≥5 days/month (30). Serum uric acid was included as a metabolic covariate because it is associated with diet, insulin resistance, and dyslipidemia and therefore may confound the association between HEI and the metabolic outcomes.

In the NHANES dataset, race/ethnicity and the ratio of family income to poverty (PIR) were additionally included as covariates. In the external validation cohort, race information was not available and socioeconomic status (SES) was used as an approximate socioeconomic indicator. Because the two cohorts differed in sampling frame, calendar period (NHANES 2005–2020 vs. hospital cohort 2024–2025), and dietary context, analyses were conducted separately rather than pooled, and no direct quantitative comparison between cohorts was intended.

### 2.6 Statistical analyses

All statistical analyses were conducted using R (version 4.4.3). Continuous variables were expressed as means ± standard deviations (SDs), and categorical variables as frequencies and percentages. Group differences were assessed using *t*-tests or analysis of variance for continuous variables and chi-square tests for categorical variables.

Given the wide distribution ranges of HEI and SII, natural logarithmic transformations were applied prior to regression and mediation analyses.

Multivariable linear regression models were used to evaluate the associations between HEI and metabolic aging markers (HOMA-IR and AIP), as well as between SII and metabolic outcomes. Three models were constructed with progressive adjustment: Model 1 adjusted for age and sex (and race in NHANES only); Model 2 further adjusted for marital status, obesity, socioeconomic indicators (PIR in NHANES and SES in validation cohort), and total energy intake; Model 3 additionally adjusted for smoking status, alcohol consumption, hypertension, non-HDL cholesterol, and serum uric acid levels.

Restricted cubic spline (RCS) analyses with three knots (at the 10th, 50th, and 90th percentiles of HEI) were performed to assess potential non-linear dose–response relationships.

Mediation analyses were conducted using a counterfactual-based framework implemented in the R package mediation to estimate the extent to which SII statistically accounted for the observed HEI-outcome associations. Because both cohorts were cross-sectional, these analyses should be interpreted as statistical decomposition of associations rather than proof of causal mediation. The proportion mediated was calculated as the ratio of the indirect effect to the total effect. Bootstrapping with 1,000 resamples was used to estimate confidence intervals (Table 1).

**Table 1:** Baseline characteristics of US adults in NHANES 2005–2020 according to quartiles of healthy eating index-2015.

Variable	Healthy eating index					
	Total	Q1	Q2	Q3	Q4	*p*-value
Total	15,314(100.00)	3,828(24.91)	3,828(25.47)	3,829(24.90)	3,829(24.71)	
Age~years	47.15(0.27)	43.18(0.35)	45.90(0.40)	48.25(0.37)	51.33(0.45)	<0.01
Sex~%						<0.01
Female	7,719(50.30)	1816(48.36)	1820(46.48)	1980(52.26)	2,103(54.20)	
Male	7,596(49.70)	2013(51.64)	2008(53.52)	1849(47.74)	1726(45.80)	
Race~%						<0.01
Non-Hispanic White	6,799(69.91)	1742(68.96)	1730(70.53)	1,683(68.94)	1,644(71.22)	
Non-Hispanic Black	3,128(9.83)	908(11.75)	834(10.35)	772(9.82)	614(7.37)	
Mexican American	2,322(8.12)	561(8.61)	573(8.40)	630(8.54)	558(6.90)	
Other Hispanic	1,412(5.17)	308(4.82)	336(4.80)	344(5.26)	424(5.82)	
Other race	1,654(6.97)	310(5.87)	355(5.92)	400(7.45)	589(8.68)	
Marital status						<0.01
Married/living with partner	9,342(64.73)	2,169(59.93)	2,335(64.27)	2,345(65.94)	2,493(68.83)	
Widowed/divorced/separated	3,241(17.85)	758(17.60)	792(18.03)	860(18.50)	831(17.27)	
Never married	2,732(17.42)	902(22.47)	701(17.70)	624(15.56)	505(13.90)	
Obesity~%						<0.01
No	9,452(62.93)	2,141(56.01)	2,302(61.26)	2,386(63.51)	2,623(71.04)	
Yes	5,862(37.07)	1,687(43.99)	1,526(38.74)	1,443(36.49)	1,206(28.96)	
Family PIR	3.08(0.03)	2.74(0.05)	3.04(0.04)	3.12(0.04)	3.44(0.04)	<0.01
Energy intake~kcal	2199.00(10.77)	2278.80(23.94)	2272.07(20.02)	2181.02(20.48)	2061.35(19.55)	<0.01
Smoking behavior~%						<0.01
Never	8,393(54.67)	1910(50.66)	1977(51.11)	2,168(57.61)	2,338(59.44)	
Former	3,822(25.94)	830(22.68)	940(26.08)	982(25.43)	1,070(29.58)	
Now	3,100(19.39)	1,089(26.66)	911(22.80)	679(16.96)	421(10.98)	
Alcohol consumption~%						<0.01
Never	1937(9.81)	416(8.66)	414(8.15)	504(10.79)	603(11.68)	
Former	2,111(11.72)	557(13.11)	542(12.29)	543(11.66)	469(9.82)	
Mild	5,541(38.83)	1,256(35.29)	1,310(36.59)	1,343(37.44)	1,632(46.10)	
Moderate	2,503(18.22)	644(18.16)	664(18.57)	616(18.79)	579(17.33)	
Heavy	3,223(21.42)	956(24.77)	898(24.39)	823(21.33)	546(15.07)	
Hypertension						0.46
No	8,937(62.64)	2,282(63.77)	2,244(61.87)	2,216(61.70)	2,195(63.23)	
Yes	6,378(37.36)	1,547(36.23)	1,584(38.13)	1,613(38.30)	1,634(36.77)	
HOMA-IR	3.66(0.06)	4.10(0.13)	3.93(0.15)	3.56(0.10)	3.05(0.10)	<0.01
SII	525.83(3.85)	538.56(5.61)	543.81(7.02)	524.47(7.01)	495.83(5.76)	<0.01
AIP	−0.06(0.00)	−0.02(0.01)	−0.05(0.01)	−0.07(0.01)	−0.12(0.01)	<0.01
Non-HDL~ mg/dL	138.18(0.54)	139.11(1.03)	139.13(0.87)	139.20(0.95)	135.25(0.98)	<0.01
Serum uric acid~μmol/L	325.72(1.03)	325.95(1.72)	327.26(1.97)	327.90(1.94)	321.71(1.69)	0.02

Data are presented as mean (standard error) for continuous variables and n (%) for categorical variables. *p*-values were calculated using *t*-tests or ANOVA for continuous variables and chi-square tests for categorical variables. HEI, Healthy Eating Index; HOMA-IR, Homeostasis Model Assessment of Insulin Resistance; AIP, Atherogenic Index of Plasma; SII, Systemic Immune-Inflammation Index; PIR, ratio of family income to poverty; NHDL, non-HDL cholesterol. Statistical significance was set at *P* < 0.05.

To examine reproducibility, the primary analyses performed in NHANES were repeated in the external hospital-based cohort using the same modeling framework. We interpreted concordant directions of association as supportive of reproducibility across settings, while recognizing that differences in dietary culture, food composition, and sampling frame limit strict cross-cultural comparability and broader generalization.

For NHANES, all analyses accounted for the complex multistage sampling design and applied appropriate sampling weights. Statistical significance was defined as a two-sided *p* < 0.05.

## 3 Results

### 3.1 Characteristics of the included population

A total of 15,314 participants from NHANES and 833 individuals from the external validation cohort were included in the analysis.

In the NHANES cohort, participants with higher HEI scores tended to be older, more likely to be female, and to have higher socioeconomic status (all *p* < 0.01). They also had lower prevalence of smoking and heavy alcohol consumption. Higher HEI was accompanied by lower HOMA-IR, SII, and AIP, indicating a more favorable metabolic and inflammatory profile at the descriptive level.

In the external validation cohort, most baseline demographic characteristics were more balanced across HEI quartiles (Table 2), although SES differed significantly. HOMA-IR, SII, and AIP still showed decreasing trends across increasing HEI categories (all *p* < 0.01), suggesting that the inverse association was also observable in this independent clinical sample.

**Table 2:** Baseline characteristics of adults undergoing health examination in Shandong, China, 2024–2025, according to quartiles of healthy eating index-2015.

Variable	Healthy eating index					
	Total	Q1	Q2	Q3	Q4	*P*-value
Total	833(100.00)	209(25.09)	208(24.97)	208(24.97)	208(24.97)	
Age~years	48.28(11.68)	47.93(11.25)	48.64(12.18)	47.29(11.14)	49.25(12.12)	0.35
Sex~%						0.30
Female	392(47.06)	89(42.58)	105(50.48)	104(50.00)	94(45.19)	
Male	441(52.94)	120(57.42)	103(49.52)	104(50.00)	114(54.81)	
Marital status						0.39
Married/living with partner	566(67.95)	138(66.03)	139(66.83)	145(69.71)	144(69.23)	
Widowed/divorced/separated	135(16.21)	37(17.70)	32(15.38)	39(18.75)	27(12.98)	
Never married	132(15.85)	34(16.27)	37(17.79)	24(11.54)	37(17.79)	
Obesity~%						0.70
No	755(90.64)	190(90.91)	192(92.31)	188(90.38)	185(88.94)	
Yes	78(9.36)	19(9.09)	16(7.69)	20(9.62)	23(11.06)	
SES	0.01(0.96)	−0.18(0.98)	−0.02(0.99)	0.08(0.96)	0.17(0.89)	<0.01
Energy intake~kcal	2050.50(412.83)	2052.93(387.87)	2083.92(443.78)	2048.58(406.55)	2016.57(411.49)	0.43
Smoking behavior~%						0.19
Never	451(54.14)	122(58.37)	123(59.13)	108(51.92)	98(47.12)	
Former	172(20.65)	41(19.62)	39(18.75)	42(20.19)	50(24.04)	
Now	210(25.21)	46(22.01)	46(22.12)	58(27.88)	60(28.85)	
Alcohol consumption~%						0.33
Never	167(20.05)	39(18.66)	42(20.19)	51(24.52)	35(16.83)	
Former	74(8.88)	23(11.00)	21(10.10)	15(7.21)	15(7.21)	
Mild	346(41.54)	85(40.67)	91(43.75)	82(39.42)	88(42.31)	
Moderate	152(18.25)	44(21.05)	33(15.87)	31(14.90)	44(21.15)	
Heavy	94(11.28)	18(8.61)	21(10.10)	29(13.94)	26(12.50)	
Hypertension						0.15
No	615(73.83)	158(75.60)	157(75.48)	159(76.44)	141(67.79)	
Yes	218(26.17)	51(24.40)	51(24.52)	49(23.56)	67(32.21)	
HOMA-IR	3.32(0.97)	3.57(0.92)	3.49(0.97)	3.18(0.98)	3.02(0.92)	<0.01
SII	880.45(243.76)	939.60(234.96)	897.17(250.50)	849.64(245.73)	835.12(231.07)	<0.01
AIP	−0.13(0.12)	−0.10(0.12)	−0.12(0.11)	−0.14(0.12)	−0.16(0.11)	<0.01
Non-HDL~ mg/dL	158.29(28.06)	158.93(28.50)	157.13(28.92)	158.22(27.31)	158.87(27.64)	0.91
Serum uric acid~μmol/L	385.43(67.12)	387.72(70.35)	387.52(66.26)	387.00(62.86)	379.49(68.87)	0.54

### 3.2 Relationship between HEI levels and risk of metabolic aging

As shown in Table 3, HEI was inversely associated with both markers of metabolic aging in a dose-dependent manner.

**Table 3:** Associations of healthy eating index-2015 with HOMA-IR and AIP in US adults from NHANES 2005–2020 and adults undergoing health examination in Shandong, China, 2024–2025.

Implicit variable	Model1	*p*-value	Model2	*P*-value	Model3	*P*-value
	β (95%CI)		β (95%CI)		β (95%CI)	
NHANES data						
HOMA-IR						
Continuous	−1.65(−2.08, −1.22)	<0.01	−0.77(−1.17, −0.37)	<0.01	−0.70(−1.10, −0.30)	<0.01
Q1	Reference					
Q2	−0.26(−0.65, 0.13)	0.19	−0.02(−0.39, 0.34)	0.91	−0.01(−0.37, 0.35)	0.96
Q3	−0.68(−1.00, −0.36)	<0.01	−0.33(−0.62, −0.04)	0.03	−0.31(−0.60, −0.02)	0.03
Q4	−1.24(−1.55, −0.92)	<0.01	−0.58(−0.86, −0.29)	<0.01	−0.52(−0.81, −0.23)	<0.01
AIP						
Continuous	−0.14(−0.16, −0.11)	<0.01	−0.08(−0.11, −0.06)	<0.01	−0.06(−0.08, −0.04)	<0.01
Q1	Reference					
Q2	−0.04(−0.06, −0.02)	<0.01	−0.02(−0.04, 0.00)	0.03	−0.02(−0.04, 0.00)	0.03
Q3	−0.05(−0.07, −0.03)	<0.01	−0.03(−0.05, −0.01)	<0.01	−0.03(−0.04, −0.01)	<0.01
Q4	−0.11(−0.13, −0.09)	<0.01	−0.07(−0.09, −0.05)	<0.01	−0.05(−0.07, −0.03)	<0.01
Validation data						
HOMA-IR						
Continuous	−1.32(−1.66, −0.98)	<0.01	−1.34(−1.68, −1.01)	<0.01	−1.31(−1.65, −0.98)	<0.01
Q1	Reference					
Q2	−0.09(−0.27, 0.09)	0.33	−0.09(−0.27, 0.09)	0.34	−0.09(−0.27, 0.09)	0.31
Q3	−0.41(−0.59, −0.22)	<0.01	−0.41(−0.59, −0.22)	<0.01	−0.40(−0.58, −0.22)	<0.01
Q4	−0.55(−0.73, −0.37)	<0.01	−0.56(−0.74, −0.38)	<0.01	−0.55(−0.73, −0.37)	<0.01
AIP						
Continuous	−0.12(−0.16, −0.08)	<0.001	−0.13(−0.16, −0.09)	<0.01	−0.12(−0.16, −0.08)	<0.01
Q1	Reference					
Q2	−0.02(−0.04, 0.00)	0.09	−0.02(−0.04, 0.00)	0.08	−0.02(−0.04, 0.00)	0.07
Q3	−0.04(−0.06, −0.01)	<0.01	−0.04(−0.06, −0.02)	<0.01	−0.04(−0.06, −0.02)	<0.01
Q4	−0.05(−0.07, −0.03)	<0.01	−0.06(−0.08, −0.04)	<0.01	−0.05(−0.08, −0.03)	<0.01

In the NHANES cohort, higher HEI was significantly associated with lower HOMA-IR and AIP across all models. After full adjustment (Model 3), each unit increase in HEI was associated with lower HOMA-IR (*β* = −0.70, 95% CI: −1.10 to −0.30, *p* < 0.01) and lower AIP (*β* = −0.06, 95% CI: −0.08 to −0.04, *p* < 0.01). Quartile analyses also showed an inverse gradient, with the lowest estimates in Q4.

Similar inverse associations were observed in the external validation cohort. HEI remained significantly inversely associated with HOMA-IR (*β* = −1.31, 95% CI: −1.65 to −0.98, *p* < 0.01) and AIP (*β* = −0.12, 95% CI: −0.16 to −0.08, *p* < 0.01) after full adjustment. The larger coefficients in the validation dataset should be interpreted cautiously because the hospital-based cohort differed from NHANES in sampling frame, calendar period, and dietary context.

### 3.3 Relationship between SII levels and risk of metabolic aging

Table 4 shows positive associations between SII and the metabolic aging indicators.

**Table 4:** Associations of SII with HOMA-IR and AIP in US adults from NHANES 2005–2020 and adults undergoing health examination in Shandong, China, 2024–2025.

Implicit variable	Model1	*P*-value	Model2	*P*-value	Model3	*P*-value
	β (95%CI)		β (95%CI)		β (95%CI)	
NHANES data						
HOMA-IR						
Continuous	0.74(0.55, 0.93)	<0.01	0.37(0.20, 0.54)	<0.01	0.34(0.17, 0.51)	<0.01
Q1	Reference					
Q2	0.39(0.10, 0.69)	0.01	0.22(−0.04, 0.49)	0.10	0.23(−0.04, 0.50)	0.09
Q3	0.60(0.37, 0.84)	<0.01	0.22(0.00, 0.44)	0.05	0.23(0.01, 0.45)	0.04
Q4	0.90(0.64, 1.16)	<0.01	0.40(0.15, 0.66)	<0.01	0.35(0.10, 0.61)	0.01
AIP						
Continuous	0.05(0.04, 0.07)	<0.01	0.03(0.02, 0.05)	<0.01	0.03(0.01, 0.04)	<0.01
Q1	Reference					
Q2	0.04(0.02, 0.06)	<0.01	0.03(0.01, 0.05)	0.01	0.02(0.00, 0.04)	0.03
Q3	0.07(0.04, 0.09)	<0.01	0.04(0.02, 0.07)	<0.01	0.03(0.02, 0.05)	<0.01
Q4	0.08(0.06, 0.10)	<0.01	0.05(0.03, 0.07)	<0.01	0.04(0.02, 0.05)	<0.01
Validation data						
Continuous	1.38(1.15, 1.60)	<0.01	1.33(1.10, 1.56)	<0.01	1.30(1.08, 1.53)	<0.01
Q1	Reference					
Q2	0.35(0.18, 0.53)	<0.01	0.35(0.18, 0.53)	<0.01	0.35(0.17, 0.53)	<0.01
Q3	0.64(0.47, 0.82)	<0.01	0.62(0.45, 0.80)	<0.01	0.61(0.43, 0.79)	<0.01
Q4	0.98(0.81, 1.16)	<0.01	0.95(0.77, 1.13)	<0.01	0.93(0.75, 1.11)	<0.01
AIP						
Continuous	0.25(0.22, 0.27)	<0.01	0.23(0.21, 0.25)	<0.01	0.23(0.21, 0.25)	<0.01
Q1	Reference					
Q2	0.06(0.04, 0.08)	<0.01	0.06(0.04, 0.07)	<0.01	0.06(0.04, 0.07)	<0.01
Q3	0.11(0.09, 0.12)	<0.01	0.10(0.08, 0.12)	<0.01	0.10(0.08, 0.12)	<0.01
Q4	0.18(0.16, 0.20)	<0.01	0.17(0.15, 0.18)	<0.01	0.16(0.15, 0.18)	<0.01

In the NHANES dataset, higher SII was significantly associated with increased HOMA-IR (*β* = 0.34, 95% CI: 0.17–0.51, *p* < 0.01) and AIP (*β* = 0.03, 95% CI: 0.01–0.04, *p* < 0.01) after multivariable adjustment. A clear dose–response relationship was observed across SII quartiles, supporting a graded effect of systemic inflammation on metabolic dysfunction.

These associations were also observed in the external validation cohort. Fully adjusted analyses showed that SII was associated with HOMA-IR (*β* = 1.30, 95% CI: 1.08–1.53, *p* < 0.01) and AIP (*β* = 0.23, 95% CI: 0.21–0.25, *p* < 0.01). The larger coefficients in this cohort may reflect differences in case mix and measurement context rather than a stronger biological effect alone.

### 3.4 Relationship between HEI levels and SII

The relationship between HEI and SII is presented in Table 5.

**Table 5:** Associations of healthy eating index-2015 with SII in US adults from NHANES 2005–2020 and adults undergoing health examination in Shandong, China, 2024–2025.

Implicit variable	Model1	*P*-value	Model2	*P*-value	Model3	*P*-value
	β (95%CI)		β (95%CI)		β (95%CI)	
SII						
NHANES data						
Continuous	−0.16(−0.20, −0.12)	<0.01	−0.13(−0.17, −0.09)	<0.01	−0.12(−0.16, −0.08)	<0.01
Q1	Reference					
Q2	−0.01(−0.04, 0.02)	0.54	0.00(−0.03, 0.03)	0.96	0.00(−0.03, 0.03)	0.94
Q3	−0.05(−0.08, −0.03)	<0.01	−0.04(−0.07, −0.01)	0.01	−0.04(−0.06, −0.01)	0.01
Q4	−0.12(−0.15, −0.08)	<0.01	−0.09(−0.12, −0.06)	<0.01	−0.08(−0.11, −0.05)	<0.01
Validation data						
Continuous	−0.31(−0.40, −0.21)	<0.01	−0.31(−0.40, −0.21)	<0.01	−0.30(−0.40, −0.21)	<0.01
Q1	Reference					
Q2	−0.05(−0.10, −0.00)	0.05	−0.05(−0.10, −0.00)	0.05	−0.05(−0.10, −0.00)	0.04
Q3	−0.10(−0.15, −0.05)	<0.01	−0.10(−0.15, −0.05)	<0.01	−0.10(−0.15, −0.05)	<0.01
Q4	−0.13(−0.18, −0.08)	<0.01	−0.13(−0.18, −0.08)	<0.01	−0.13(−0.18, −0.08)	<0.01

In the NHANES cohort, HEI was significantly inversely associated with SII in both continuous and categorical analyses. After full adjustment, HEI remained negatively associated with SII (*β* = −0.12, 95% CI: −0.16 to −0.08, *p* < 0.01), with a clear decreasing trend across quartiles.

This inverse relationship was also observed in the external validation cohort, where HEI was negatively associated with SII (*β* = −0.30, 95% CI: −0.40 to −0.21, *p* < 0.01). The consistency in direction supports replication of the association in an independent clinical setting.

### 3.5 Non-linear relationship between HEI and risk of metabolic aging

**Figure 2:**
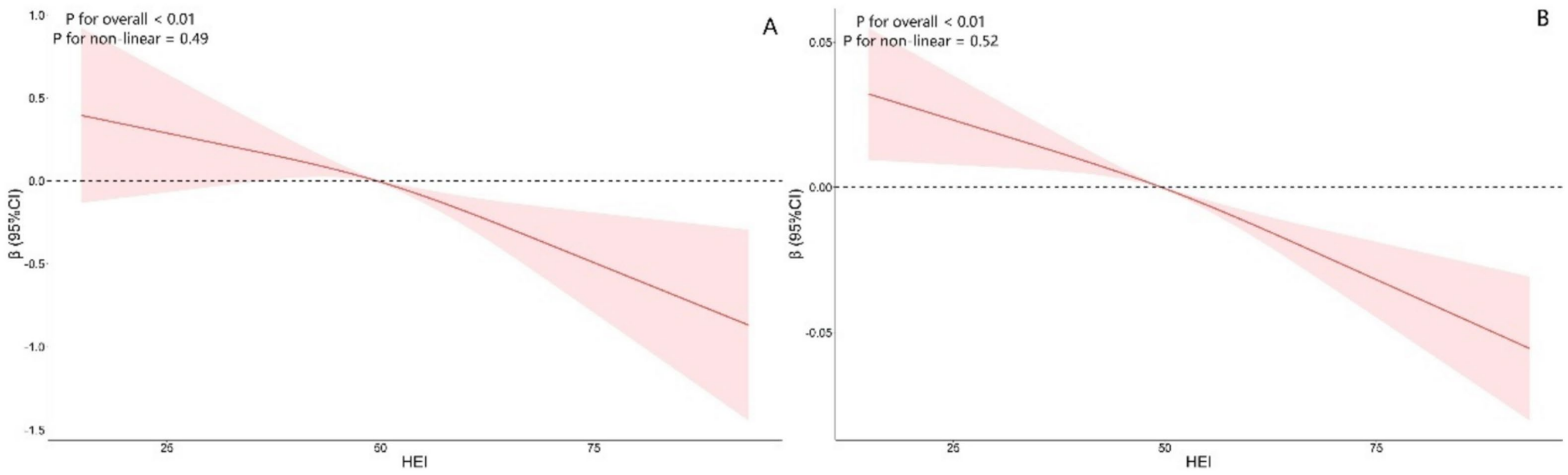
Restricted cubic spline showing the association of HEI with HOMA-IR **(A)** and AIP **(B)** in U. S. adults from NHANES 2005–2020.

**Figure 3:**
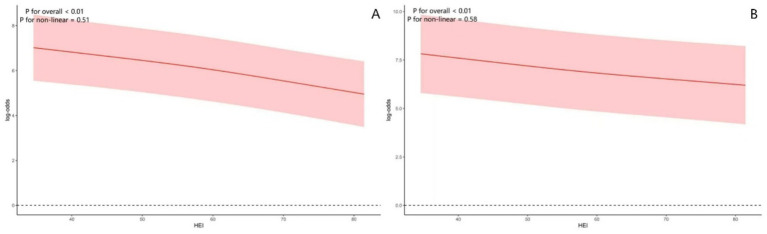
Restricted cubic spline showing the association of HEI with HOMA-IR **(A)** and AIP **(B)** in adults undergoing health examination in Shandong, China, 2024–2025.

Restricted cubic spline analyses were performed to evaluate the potential non-linear relationship between HEI and metabolic aging markers (Figures 2, 3).

In the NHANES cohort, HEI exhibited a significant inverse association with both HOMA-IR (P for overall < 0.01) and AIP (P for overall < 0.01), with no evidence of non-linearity (P for non-linearity = 0.49 and 0.52, respectively). These findings indicate that the associations between dietary quality and metabolic aging were predominantly linear across the full range of HEI values.

Consistent results were observed in the external validation cohort (Figure 3). HEI remained inversely associated with HOMA-IR (P for overall < 0.01; P for non-linearity = 0.51) and AIP (P for overall < 0.01; P for non-linearity = 0.58). The spline curves demonstrated a similar linear downward trend, further confirming that improvements in dietary quality were associated with progressive reductions in metabolic risk without a detectable threshold effect.

### 3.6 Mediation effects

**Figure 4:**
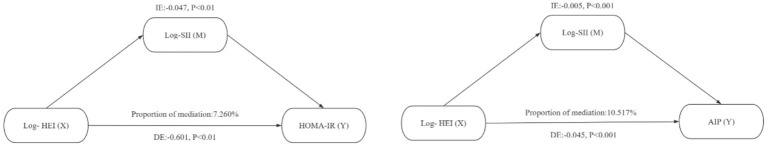
Statistical mediation analyses for the association of HEI with metabolic aging markers through SII in U. S. adults from NHANES 2005–2020.

**Figure 5:**
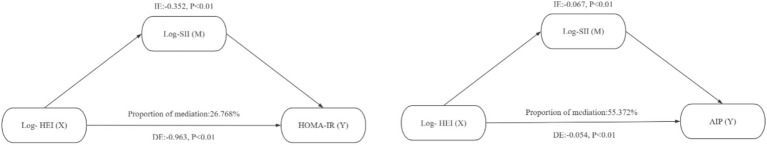
Statistical mediation analyses for the association of HEI with metabolic aging markers through SII in adults undergoing health examination in Shandong, China, 2024–2025.

Mediation analyses were conducted to evaluate whether systemic inflammation, as measured by SII, statistically partially accounted for the association between HEI and metabolic aging (Figures 4, 5).

In the NHANES cohort, SII statistically accounted for 7.260% of the total HEI-HOMA-IR association [indirect effect (IE) = −0.047, *p* < 0.01; direct effect (DE) = −0.601, *p* < 0.01]. Similarly, SII statistically accounted for 10.517% of the HEI-AIP association (IE = −0.005, *p* < 0.001; DE = −0.045, *p* < 0.001).

In the external validation cohort, SII also statistically accounted for part of the association (Figure 5). For HOMA-IR, the indirect effect was −0.352 (*p* < 0.01), with a proportion mediated of 26.768%, while the direct effect remained significant (DE = −0.963, *p* < 0.01). For AIP, SII statistically accounted for 55.372% of the association (IE = −0.067, *p* < 0.01; DE = −0.054, *p* < 0.01). These larger mediated proportions should be interpreted cautiously given the smaller sample size and the clinical nature of the validation cohort.

Overall, these findings suggest that systemic inflammation, as captured by SII, may represent one pathway related to the observed HEI-metabolic aging association, although the cross-sectional design precludes causal inference.

## 4 Discussion

In this cross-sectional study, higher HEI scores were associated with lower HOMA-IR and AIP in both NHANES and an external hospital-based cohort from China. SII statistically partially accounted for part of these associations. These findings support an inverse relationship between overall diet quality and metabolic aging-related markers, while also indicating that systemic inflammation may be one relevant correlate.

The inverse associations between HEI and the metabolic markers are consistent with previous studies on diet quality and cardiometabolic health (31–33). Diets with higher HEI scores generally include more whole grains, fruits, vegetables, and unsaturated fats and less refined carbohydrates and processed foods, which may be related to better insulin sensitivity and lipid metabolism (34–36). However, because our study was cross-sectional, these mechanisms should be interpreted as biologically plausible explanations rather than confirmed causal pathways.

Importantly, the associations remained after multivariable adjustment. Restricted cubic spline analyses suggested approximately linear inverse relationships between HEI and both HOMA-IR and AIP within the observed range, implying that better diet quality was associated with progressively lower metabolic risk markers. Nevertheless, the effect size for AIP was modest, especially in NHANES, and its clinical meaning is better viewed as a population-level shift in a lipid-related risk marker rather than a large individual-level effect.

A strength of the study is that the same analytical framework was examined in an external Chinese hospital-based cohort. This does not establish full cross-cultural comparability or universal generalizability, because food composition, cooking methods, health-care-seeking behavior, and the distribution of HEI components likely differ between the United States and China. Rather, the external cohort provides a more limited form of validation by showing that the direction of the association was reproducible in a distinct non-U. S. clinical setting.

An important methodological consideration is the difference in calendar time between the two datasets. The NHANES data span 2005–2020, whereas the external hospital-based cohort was collected in 2024–2025. Changes over time in dietary patterns, healthcare access, and population health profiles may influence the observed associations. Therefore, the external cohort should be interpreted as a temporal and contextual replication of the direction of associations rather than a contemporaneous validation. This temporal discrepancy further supports our decision to avoid direct quantitative comparisons and instead focus on consistency in association patterns.

The finding that SII statistically partially accounted for the HEI–metabolic aging association is consistent with the broader inflammation-metabolism hypothesis. Several mechanisms may underlie this pattern: (1) dietary fiber and polyphenols may modulate gut microbiota and increase SCFA production (37–39); (2) n-3 polyunsaturated fatty acids may inhibit pro-inflammatory cytokine release and mitigate chronic low-grade inflammation (38); and (3) higher-quality diets may reduce oxidative stress and inflammatory signaling (40–42). At the same time, SII is only one composite inflammatory index, and we did not compare it with classic inflammatory markers such as high-sensitivity C-reactive protein, IL-6, or TNF-*α*.

From a clinical and public health perspective, these findings suggest that better diet quality may be linked to more favorable metabolic profiles and lower systemic inflammation. SII may be a pragmatic marker for risk stratification in settings where complete blood count data are readily available. However, these implications should be interpreted cautiously, because intervention benefit cannot be inferred from cross-sectional associations.

Several limitations should be acknowledged. First, the cross-sectional design precludes conclusions about temporality or causality. Second, dietary intake was assessed using 24-h recalls, which are susceptible to recall error. In the external cohort, HEI-2015 was used as a harmonized scoring framework to reproduce the exposure definition, but this U. S.-based index may not fully reflect Chinese dietary culture. Third, the validation cohort was hospital-based and therefore is not representative of the general Chinese population. Fourth, residual confounding remains possible, and we were unable to account for some potentially important factors, including classic inflammatory markers (e.g., high-sensitivity C-reactive protein, IL-6, TNF-*α*), detailed medication use, physical activity, sleep, stress, and other behavioral or clinical factors. Fifth, while serum uric acid was adjusted for as a metabolic covariate, overadjustment cannot be completely excluded. Finally, SII does not capture all dimensions of inflammation. In addition, the difference in calendar periods between NHANES (2005–2020) and the validation cohort (2024–2025) may introduce temporal heterogeneity related to secular trends in diet and metabolic health.

Future studies should use prospective designs, incorporate multi-marker inflammatory panels, and examine cross-cultural comparability of diet-quality metrics. Standardization or calibration of HEI-like tools across dietary cultures, together with food-group-level sensitivity analyses, may help clarify which aspects of diet quality are most relevant to metabolic aging in different populations.

In conclusion, higher HEI scores were associated with lower metabolic aging-related markers in both cohorts, and SII statistically partially accounted for these associations. The external cohort supports reproducibility of the direction of the findings in a distinct clinical setting, but differences in dietary culture and the cross-sectional design mean that broad causal or universal generalization should be avoided.

## 5 Conclusion

This study indicates that higher adherence to HEI-2015 was associated with lower HOMA-IR and AIP, and that SII statistically partially accounted for these associations. Similar directional findings were observed in an external hospital-based cohort from China. These results support the relevance of diet quality and systemic inflammation to metabolic aging-related markers, while also underscoring the need for prospective and culturally calibrated studies.

## Data Availability

The raw data supporting the conclusions of this article will be made available by the authors, without undue reservation.
